# Reply to Lemieux et al.: Integration does not preclude essentiality of mitochondrial glycerol-3-phosphate dehydrogenase

**DOI:** 10.1073/pnas.2613403123

**Published:** 2026-06-01

**Authors:** Léa Herpe, Mélanie Aminot, Nicolas Pichaud

**Affiliations:** ^a^New Brunswick Centre for Precision Medicine, Moncton, NB E1C8X3, Canada; ^b^ Department of Chemistry and Biochemistry, Université de Moncton, Moncton, NB E1A 3E9, Canada

Lemieux et al. (1) raise concerns about our recently published study supporting the essential role the mitochondrial glycerol-3-phosphate dehydrogenase (mtG3PDH) in mitochondrial bioenergetic and redox status of *Drosophila* mutants (GPO1) (2). Here, we provide responses and perspective solutions to these concerns.

mtG3DPH operates within an integrated metabolic network. Certainly, the observed organismal phenotypes from mtG3PDH disruption reflect direct and indirect effects. However, such integration does not preclude functional essentiality. Our study did not aim to resolve all mechanistic contributions of mtG3PDH within this network, but rather to provide a focused demonstration of its functional importance in a genetic loss-of-function model. In this context, removal of mtG3PDH leads to pronounced impairments in organismal performance and survival. Further work, including pathway-level and interactome analyses, will be required to disentangle its mechanistic contributions.

Regarding the quantification of the contribution to oxygen consumption, we acknowledge an error in the description of the calculation in the supplementary material. Calculations were, however, conducted using the proper formula (CI + CII + mtG3PDH − OXPHOS – CI + CII − OXPHOS)/CI + CII − OXPHOS, representing G3P contribution ratio to OXPHOS. The reduction in glycerol-3-phosphate-supported respiration in GPO1 flies remains valid and is supported by the raw respiration data.

**Fig. 1.**
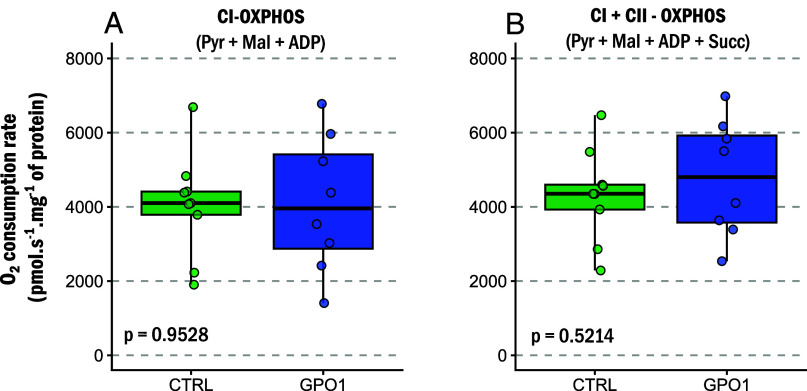
Oxygen consumption rate during CI-OXPHOS (*A*) and CI+CII-OXPHOS (*B*). Normality and homoscedasticity were checked via Shapiro–Wilk and Fisher’s tests, respectively, followed by Student’ *t* test.

The expression of ATP yield as “1.5 ATP per FADH_2_” reflects a theoretical framework rather than a physiological constant. In addition, errors in ATP production units and O_2_ conversion factors were identified and corrected shortly after publication. A revised version was made available prior to receipt of this correspondence. While these corrections affect absolute ATP/O values, they do not alter the comparative framework of our analysis. Our conclusions are based on consistent differences in oxygen consumption and ATP production measured under identical experimental conditions, rather than on absolute ATP/O values. In this context, both parameters are reduced in GPO1 flies (by 60% for ATP production and 33% for O_2_ consumption). Reanalysis including all data confirms robustness of this conclusion with no change in statistical outcome (*P* < 0.001) and no evidence for compensatory increases in CI-OXPHOS or CI+CII-OXPHOS (Fig. 1 *A* and *B*).

Concerning H_2_O_2_ emission measurements, non-phosphorylating conditions and the use of inhibitors indeed do not reflect physiological states. Our objective was to assess substrate-associated H_2_O_2_ emission under controlled bioenergetic conditions, maximizing membrane potential and revealing intrinsic differences in redox behavior. Notably, conditions without inhibitors (figure 3 H and I in ref. 2) also show reduced H_2_O_2_ emission in GPO1 flies, supporting a direct contribution of mtG3PDH to mitochondrial redox balance, which is in accordance with several publications (3–5). In vivo approaches will be required to establish physiological relevance.

Finally, while glycerol-3-phosphate has long been used as a substrate in mitochondrial studies, the functional importance of mtG3PDH itself, particularly at the organismal level, remains unresolved and is strongly context dependent (6–10). Our results show that disruption of this enzyme leads to significant mitochondrial and organismal consequences in *Drosophila*, demonstrating that mtG3PDH can become functionally essential depending on biological context.

We believe that continued work across model systems will be essential to resolve the context-dependent roles of mtG3PDH in organismal bioenergetics.
